# Age-related nest-site segregation in a solitary nesting population of white stork *Ciconia Ciconia*

**DOI:** 10.1186/s12983-025-00574-2

**Published:** 2025-08-04

**Authors:** Joanna T. Bialas, Irene Gaona-Gordillo, Łukasz Dylewski, Marcin Tobolka

**Affiliations:** 1 Department of Zoology, Poznań University of Life Sciences, Wojska Polskiego 71C, 60-625 Poznań, Poland; 2 Department of Biology, Behavioural Ecology, Ludwig Maximilians University of Munich, Planegg‐Martinsried, Munich, Germany

**Keywords:** Public information, Breeding habitat, Nest size, Age-related competition, Habitat selection

## Abstract

**Background:**

Habitat selection plays a crucial role in avian reproductive success, with nest-site characteristics and individual traits influencing breeding outcomes. This study investigates the relationship between breeder’s age and nest-site selection in a population of white storks *Ciconia ciconia*, a long-lived, site-faithful species nesting solitarily in Western Poland. Using data collected from 2006 to 2024 on ring recoveries, nest dimensions, productivity, and habitat characteristics, we analyzed the age at which birds were first recorded on specific nests.

**Results:**

Results revealed that older individuals were more likely to occupy larger nests and nests with higher productivity in previous years, suggesting the use of public information and a preference for high-quality sites. Interestingly, nest surface area, rather than height, was correlated with breeder’s age, while land cover surrounding the nest had a sex-specific effect. Older individuals also appeared to prefer nests with higher occupancy rates. Older females avoided human-altered habitats, whereas no such relationship was observed for males. Contrary to expectations, age-related differences were not associated with preferred foraging habitats like meadows and pastures.

**Conclusions:**

These findings highlight that age-based segregation in nest-site selection reflects experience and competition, with older birds optimizing breeding success by leveraging key environmental and social cues. The results suggest that younger individuals may occupy suboptimal habitats, possibly due to competition or inexperience. Future studies should explore the role of site fidelity, age-assortative mating, and anthropogenic influences, such as supplementary feeding, to fully understand the dynamics of nest-site selection in white storks.

**Supplementary Information:**

The online version contains supplementary material available at 10.1186/s12983-025-00574-2.

## Background

Selecting a breeding habitat is a crucial determinant of avian reproductive success. The habitat must offer protection from predators but also supply resources necessary for rearing offspring, directly influencing the reproductive output of a breeding pair [1]. While population-level patterns of habitat selection have been extensively studied, the role of intrinsic factors such as age and sex remains less explored. These factors influence breeding site choice through experience, dominance, and sexual selection.

Age plays a particularly important role. It is closely linked to site fidelity [2–4], and timing of arrival at breeding grounds [5–8], both of which influence habitat selection and access to high-quality sites. This pattern aligns with the ideal despotic distribution, where dominant, often older individuals monopolize the best territories, relegating subordinates to suboptimal areas [9]. Dominance is typically linked to age and plays a crucial role in habitat selection during the breeding season [10]. In migratory species, arrival timing—and by extension, access to optimal nests [11]—can also be shaped by migratory strategy [12].

Assessing age effects on habitat selection typically requires long-term data of individually-marked birds. Yet many ornithological systems only allow categorizing birds into broad age groups (e.g., first-year vs. adult), which often does not suffice for detailed research into breeding habitat selection. This limitation not only narrows the scope of potential conclusions but also imposes significant constraints on the findings. This is especially problematic in long-lived species, where age-related effects may accumulate slowly and go undetected. Studies on age segregation, tend to focus on non-breeding periods and simplified age classes, comparing only juveniles or immature birds with adults [13–15], but evidence exists that birds exhibit age-based spatial segregation both outside and during the breeding [16–20]. In northern wheatear *Oenanthe oenanthe*, yearlings tend to settle in lower-quality breeding habitats, although the study primarily highlighted differences between just two age classes: yearlings and adults [20].

In colonial breeders of long-lived birds like Australian gannet *Morus serrator*, white stork *Ciconia ciconia* or great cormorant *Phallacrocorax carbo sinensis* younger birds typically nest on the colony periphery [3, 21, 22], which may expose them to higher predation risk and reduced breeding success [23, 24]. However, once age is controlled for, nest location within the colony may not predict success [3, 22]. In eider ducks *Somateria mollissima* females favor concealed, central nest sites, though many optimal sites remain unused, suggesting that age-related experience, rather than direct competition, shapes nest selection [25, 26].

Many features of the nesting site could serve as cues in its selection, including physical characteristics of nests in species that reuse nests from previous breeding seasons. Whether built by themselves or other individuals – nest size can signal male quality, past breeding success, or territory value [27, 28]. Further, nest size is a known predictor of reproductive success, particularly influencing clutch size [29]. Therefore, securing the largest nests often involves intense competition, necessitating early arrival at the breeding site in migratory species [30]. The size of the nest serves as a signal to females of the male’s condition or quality, whether through early arrival, nest enlargement, or as an aspect of courtship displaying their ability to provide nesting material. It also suggests that the nest is located in a territory where previous breeding attempts were successful, which is also an important cue when birds are making decision of nest selection [31].

In species that reuse nests, their size may also function as ‘public information’, signaling previous reproductive success or frequency of occupancy [32, 33]. A growing body of evidence supports the use of public information by birds in various contexts [34, 35]. Consequently, birds may also utilize the breeding success of conspecifics as a cue for nest-site selection in subsequent years [36, 37] or they may sample the environment in a prior season, based on factors such as food availability in temporally heterogeneous environments [38, 39].

The white stork offers a strong model for studying these dynamics. It is long-lived and highly site-faithful [4]. In parts of its range, it nests solitarily on human-made structures [40], and its nests can grow considerably in size over the years [31, 32]. Researchers have extensively examined how this species selects habitats based on land cover, land use, nesting structures, and nest size preferences, addressing the impacts of increasing anthropogenic pressures [32, 40–43]. Differences in nest-site fidelity, predominantly influenced by age and past breeding success, have been documented [3, 4]. Notably, studies focusing on the Western European and North African populations of white storks have emphasized the significance of nest size [32, 44–46]. It has been observed that larger nests, which correlate with more years of occupancy, tend to be selected earlier in the season and can support larger clutch sizes. However, the total number of chicks fledged does not necessarily correlate with nest size. Interestingly, the breeder age was not correlated with initial nest size in a previous study [32], despite many studies showing that older storks are the first to arrive, while younger storks arrive later [7, 8].

Although the patterns of nest-site selection seem to be relatively well-documented, the specific effects of age (i.e., experience) on these choices remain unclear. We observe that while some nests are frequently occupied, others are seldom used, suggesting that these less popular nests are suboptimal for breeding. Consequently, these nests often experience higher rates of failure and therefore abandonment, as indicated by our current studies [4]. This research aims to elucidate the patterns of nest-site selection based on the age of individuals by highlighting potential cues utilized by breeding white storks, such as nest size and productivity. Contrary to the colonially nesting storks, the population studied here is territorial and nests solitarily. This difference might lead to distinct patterns of nest-site occupation, as factors like territoriality are known to significantly affect distribution.

We hypothesize that older individuals preferentially occupy nests associated with higher territory quality, as indicated by nest size, previous-year productivity, occupation rate, and surrounding land cover. By leveraging a large, individually-marked population of white storks and long-term data, we aim to test for age-related patterns in nest-site selection in a solitary, migratory population.

## Methods

**Fig. 1:**
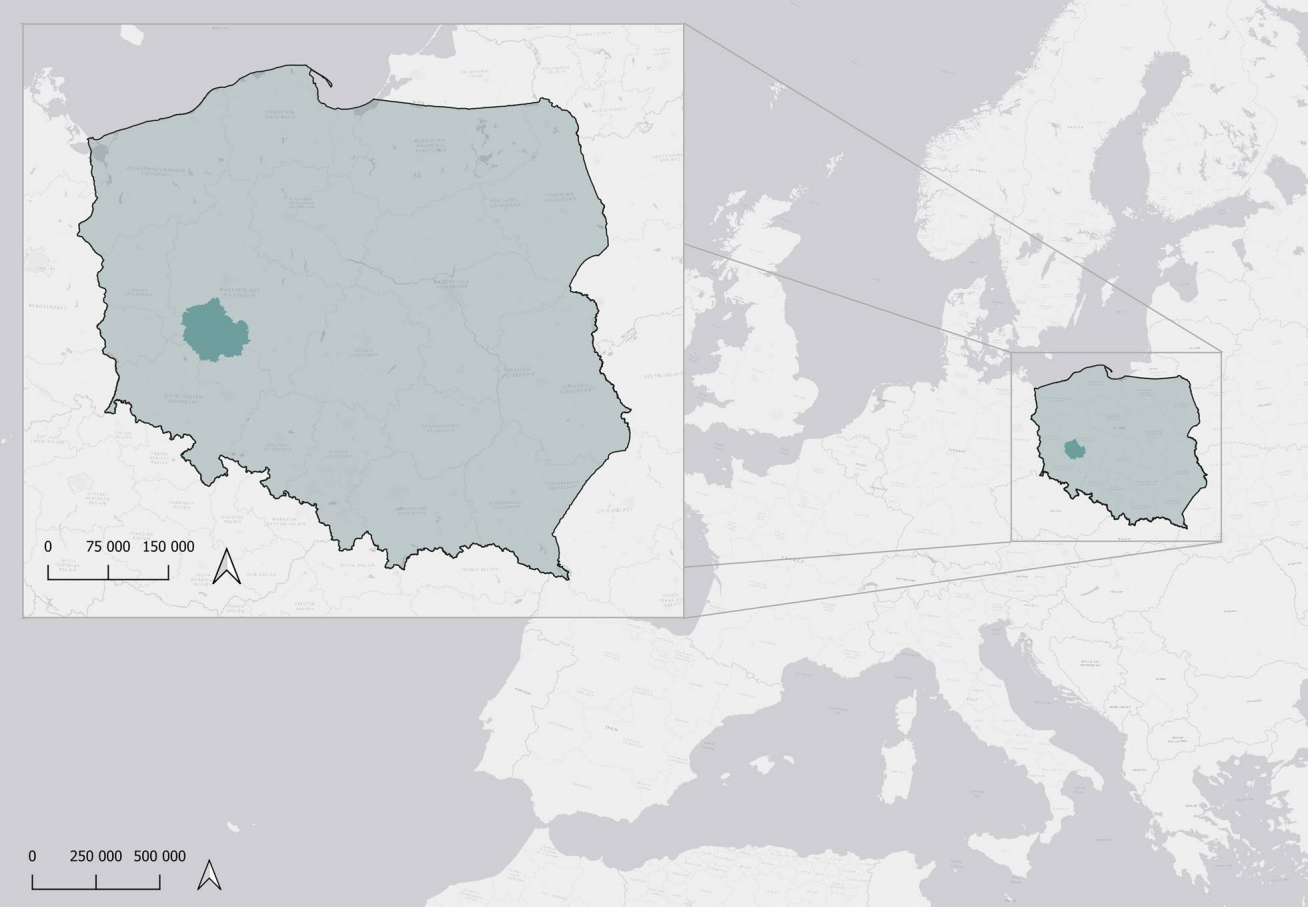
Location of the study area (dark blue) within Europe (background map) and Poland (light blue, front map)

Between 2006 and 2024, we collected data on ring recoveries, nest measurements, and census figures within the former Leszno Province in Western Poland (51°50′27.9″N 16°34′32.0″E), covering an area of 4,435 km^2^ (Fig. 1). This region is predominantly agricultural with 54% arable fields, complemented by meadows (7%), pastures (less than 1%), human settlements (10%), forests (17%), and other land uses (11%). White storks in this area are primarily solitary nesters, although in prime habitats like river valleys or near lakes, clusters of up to five active nests have been observed [47].

The majority of the data were collected by the authors, with additional ring recoveries reported through the Polish Ringing Scheme database (http://www.stornit.gda.pl/oop_en.php), contributed by volunteers. Each nest detected in previous years was recorded with its geographical coordinates and assigned to a unique nest identity (NestID). Newly built and detected nests (ranging from 2 to 25 per year, as reported by [48] were added to the existing database each season. We aimed to check all known nests and gather ring recovery data primarily at the start of the breeding season (from late March to May), which facilitated the sex determination of the storks by observing their positioning during mounting [49]. Each inspected pair occupying the nest was checked for rings on the tibiae and tarsi. Rings were deciphered either through direct observation with spotting scopes or by photographing them with a telephoto lens. We successfully read nearly every ring found (n = 701 ring recoveries from 2007 to 2024) on breeding storks. We read ring numbers of 239 unique individuals on 225 unique nests.

In late June, during chick banding, we measured occupied, successful (at least until chick-banding), and accessible nests (the number varied each year). Due to logistical constrains, we measured nest dimensions at the chick-banding stage rather than during the pre-breeding stage. We assume that nest dimensions at both stages are correlated, which is partially supported by a previous study [32]. By the first half of July, we documented the final breeding outcomes for each pair, including those with rings identified early in the season. We used a standard census method to count the number of fledglings able to stand on the nest and considered capable of flight [50].

For each recorded instance of a ringed bird occupying a nest in a given year, we estimated its age (calendar year of life) using the formula: Year of the record—Year of ringing + 1. This calculation applies to all except one individual that was banded in its second year of life; typically, birds were ringed as chicks in nests. Birds banded as adults were excluded as it was impossible to calculate their exact age.

### Spatial analysis

We obtained Corine Land Cover (CLC) datasets from the website of the Chief Inspectorate of Environmental Protection (2006–2018) for data on land cover (see Bialas et al. 2020 for details). Land cover was calculated in 2500 m radius buffers around each nest, using a processing tool in QGIS [4, 51]. For the land cover, we used Corine Land Cover datasets in 2006 for nests existing from 2006–2011, 2012 for nests existing in 2012–2017, and 2018 for nests existing in 2017–2024. We used the following classes: 1) areas greatly altered by human, which include continuous urban fabric, discontinuous urban fabric, industrial or commercial units, road and rail networks and associated land, airports, mineral extraction sites, dump sites, construction sites, green urban areas and sport and leisure facilities; 2) permanent crops, fruit trees and berries plantations; 3) non-irrigated arable land; 4) meadows and pastures; 5) other agricultural lands, which include complex cultivation patterns and land principally occupied by agriculture, with significant areas of natural vegetation and permanent crops; 6) forests, which include broad-leaved forest, coniferous forest, mixed forest and transitional woodland/shrub and sparsely vegetated areas; 8) inland marshes, 9) inland water. For all spatial analyses, we used QGIS 3.32.2 [52] open-source software.

### Statistical analysis

To test whether (a) the dimensions of the nest (n = 152 observations), (b) the relative productivity in the previous year (difference between mean number of fledglings in population and the number of fledglings on a particular nest in the previous year when the bird was first spotted on the nest, n = 222 observations), (c) the proportion of times a nest was occupied during 18 consecutive years (n = 276 observations), or (d) if the habitat surrounding the nest was related to the age of birds occupied or bred on a nest for the first time (n = 276 observations), we performed univariate generalized linear mixed models (GLMMs) that fitted age of the focal bird when first spotted on a nest as a response variable. To avoid overparameterization and due to sample size differences, we fitted four different models. All models assumed Poisson errors and were fitted with a log-link function. The first model (a) included the height of the nest centered on its mean (0.663 m) and the surface of the nest centered on its mean (1.825 m^2^) as covariates. We used these measurements separately to understand whether the height of the nest, the surface of the nest, or their interaction were related to the age when a bird bred on the nest for the first time. These covariates were centered to their mean to guarantee that the model intercept represented the estimated age of the bird when first spotted on nests of average height and surface. The second model (b) included the mean-centered number of chicks produced in the previous year on a specific nest as a covariate. This was to assess whether the relative productivity of a specific nest within the previous year can be used by the focal bird as an indicator of the quality of the nest. The third model (c) included the proportion of times the nest was occupied during 18 consecutive years centered on its mean (0.699) as a covariate. This was done to ensure that the model intercept represented the estimated age of the bird when first spotted on nests with the average proportion of occupancy. This proportion was used to acknowledge the existence of the territory, even though a nest had not yet been established. Therefore, even in cases where the nest did not exist throughout the entire study period, we utilized the full study period to calculate the proportion. Finally, the fourth model (d) included covariates representing the proportion of surrounding habitat covered by areas altered by humans (mean ± SD: 0.059 ± 0.056; range: 0.00–0.37), arable lands (mean ± SD: 0.593 ± 0.179; range: 0.10–0.94), and pastures (mean ± SD: 0.153 ± 0.141; range: 0.00–0.86), which have been shown as important for the white stork [40, 42]. All models further fitted sex (females vs. males) as fixed effect. To test for sex differences, we further fitted the interaction between sex and the aforementioned covariates, respectively. Parameter estimates for models (a)-(d) are presented both on the latent (Tables 1a, 2a, 3a, and 4a, respectively) and data (Tables 1b, 2b, 3b, and S4a, respectively) scales. We used the function “exp” (package base, R Core Team 2021) to back-transform estimates and associated 95% credible intervals (CIs). Importantly, most of the analyses showed no interactive sex effects (see Results section), and we thus reran the models after removing the interaction between sex and all the covariates described (see Tables S1a and S1b, S2a and S2b, and S3a and S3b, on the latent vs. data scale, respectively). Moreover, we did not detect any interactive effects between the height and the surface of the nest, and, thus, reran the model without this interaction (Tables S1a and S1b, on latent vs. data scale, respectively). Nonetheless, there was one exception. In the model that included the habitat surrounding the nest as a covariate (Table 4a, see Results section), we detected sex differences. We, therefore, reran model (d) separately for females (Tables 4b and S4b, on latent vs. data scale, respectively) and males (Tables 4c and S4c, on latent vs. data scale, respectively) aiming to understand the sex-specific effects of the habitat on the age when a bird was first spotted on a specific nest (n = 130 females vs. 146 males). We used a hypothesis-driven modeling approach rather than data-driven model selection. Models were constructed based on biologically meaningful hypotheses formulated a priori. In cases where interaction terms were not statistically supported, we fitted reduced models without these interactions to aid interpretation of the main effects. This procedure was not intended as model selection but rather as a way to explore and clarify the effects of individual predictors.

All models further fitted random intercepts for bird identity to account for individual differences of birds that were observed multiple times for the first time on different nests and nest identity to account for nests that were used multiple times. Furthermore, models that included both sexes combined fitted a random intercept for brood identity as both the male and female might have been spotted for the first time on the same nest. Models additionally fitted a random intercept for year to account for temporal variation. For sample size see Tables 1–4.

While we did not formally correct for spatial autocorrelation, the spatial distribution of nests across our study area (mean distance between occupied nests = 2.0 km in 2024) and non-significant Moran’s I values calculated on model residuals suggest that spatial non-independence is unlikely to have biased our results.

Statistical analyses were performed in R 4.4.1 [53]. The GLMMs were performed using the “glmer” function (package lme4, [54]). All models were tested for overdispersion and zero inflation. The function “dispersion_glmer” was used to test for overdispersion (package blmeco, [55]), where values > 1.4 suggested severe overdispersion [55]. Additionally, the function “testZeroInflation” was used to test for zero inflation (package DHARMa, [56]). We used the “sim” function (package arm, [57]) to obtain 2000 posterior simulations of model parameters, from which we then calculated the mean (β) and associated 95% CIs for each estimated parameter. The fixed effects were statistically significant in the frequentist sense when the 95% CIs did not overlap zero.

## Results

### Dimensions of the nest

**Fig. 2:**
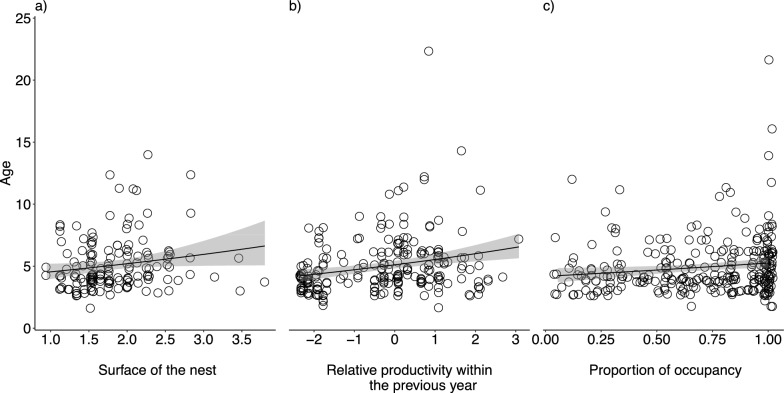
Jitter plots showing the relationships between age of a bird that bred on the nest for the first time and (**a**) the surface of the nest, (**b**) the relative productivity of a nest within the previous year, and (**c**) the proportion of times a nest was used during 18 consecutive years, with 95% confidence intervals (grey areas). Each jitter point represents the age of each individual bird

The model showed a relationship between the surface area of the nest and the age of the bird that was spotted for the first time on a nest, hereafter “age of the bird”, (main effect of Surface; Table 1a and b). However, this effect was only moderately supported as 95% CIs overlapped zero. This model further suggested that this effect did not differ between females and males (as 95% CIs for the interaction Surface × Sex overlapped zero; Table 1a). Moreover, we did not detect any effect of the height on the age of the bird (main effect of Height; Table 1a and b). To understand the overall effects of the sex, height, and surface of the nest, we reran our models after removing all the non-significant interaction terms. This model showed that the surface area was positively related to the age of the bird, but this effect did not differ between sexes (Table S1a, Fig. 2a). The back-transformed estimates of our models (Table S1b) showed that the age when birds were spotted for the first time on nests of average surface (1.825 m^2^) was of 5.11 years (intercept value Table S1b). This model further suggested that an increment of the surface of the nest by one increased the age of the bird by a factor of 1.158 (main effect of Surface; Table S1b).

**Table 1:** Estimated effect sizes and 95% credible intervals (CIs) for predictor of the age when a focal bird was spotted for the first time on a nest based on the height and the surface of the nest

	Age of the focal bird (n = 152 birds)	
	(a)	(b)
	β (95% CI)	
Intercept ^a^	1.639 (1.532, 1.747)	5.15 (4.627, 5.737)
Sex ^b^	− 0.037 (− 0.186, 0.113)	0.964 (0.830, 1.120)
*Dimensions of the nest*		
Height ^c^	0.210 (− 0.123, 0.549)	1.234 (0.884, 1.732)
Surface ^d^	0.158 (− 0.013, 0.348)	1.171 (0.987, 1.416)
Height × surface ^e^	− 0.022 (− 0.731, 0.686)	0.978 (0.481, 1.986)
*Dimensions of the nest* × *sex*		
Height × sex ^f^	− 0.125 (− 0.591, 0.374)	0.882 (0.554, 1.454)
Surface × sex ^g^	− 0.053 (− 0.343, 0.231)	0.948 (0.710, 1.260)
Height × surface × sex ^h^	0.694 (− 0.400, 1.764)	2.002 (0.670, 5.836)
	σ^2^ (95% CI)	
Bird ID	0.000 (0.000, 0.000)	1.000 (1.000, 1.000)
Nest ID	0.000 (0.000, 0.000)	1.000 (1.000, 1.000)
Brood ID	0.000 (0.000, 0.000)	1.000 (1.000, 1.000)
Year	0.005 (0.002, 0.009)	1.005 (1.002, 1.009)
Residual ^i^	0.177 (0.161, 0.196)	1.194 (1.175, 1.217)
Sample size	n	
Bird ID	124	
Nest ID	119	
Brood ID	145	
Year	14	

Predictor variables are sex (females and males), the mean-centred height of the nest (in m), the mean-centred surface of the nest (in m^2^), and their interaction. Parameters were estimated using GLMMs following a Poisson error distribution. Estimated effect sizes and 95% CIs are shown on the latent (a) and data (b) scale. Estimated effect size and 95% CIs were back-transformed from the latent scale using the function “exp” (package base, R Core Team 2024)

^a^Reference category: estimate is the age for females that were spotted for the first time on nests of average height and average surface

^b^Estimate is the difference between females (reference) and males (main effect)

^c^Effect of nests with mean-centred height (in m). Mean height of the nest was 0.663 m

^d^Effect of nests with mean-centred surface (in m^2^). Mean surface of the nest was 1.825 m^2^

^e^Estimate for the effects of the height and surface of the nest (m and m^2^, respectively; mean-centered)

^f^Estimate is the difference between females (reference) and males in the effects described in the footnote c

^g^Estimate is the difference between females (reference) and males in the effects described in the footnote d

^h^Estimate is the difference between females (reference) and males in the effects described in the footnote e

^i^Defined as ln(1/exp(βo) + 1) (following Nakagawa & Schielzeth [79])

### Relative productivity on the nest within the previous year

Relative productivity of the nest within the previous year was positively correlated with the age of the bird (main effect of Previous year relative productivity; Table 2a and b). Nonetheless, this effect did not differ between females and males (as 95% CIs for the interaction Previous year relative productivity × Sex overlapped zero; Table 2a). We thus reran the model to understand the effect of sex and the relative productivity of the nest on the previous year after removing the non-significant previous year relative productivity × Sex interaction term. This model showed that an increment in the mean-centered number of chicks produced on a nest within the previous year was positively related to the age of the bird (Table S2a, Fig. 2b). The back-transformed estimates of our model (Table S2b) showed that the age of the birds spotted for the first time on the nest with a mean-centered number of chicks in the previous year was 5.11 years (intercept value Table S2b). Specifically, increasing the mean-centered relative productivity on a nest within the previous year by one, increased the age of the focal bird by a factor of 1.082 (main effect of Previous year relative productivity; Table S2b).

**Table 2:** Estimated effect sizes and 95% credible intervals (CIs) for predictors of the age when a focal bird was spotted for the first time on a nest based on the relative productivity of a nest within the previous year

	Age of the focal bird (n = 222 birds)	
	(a)	(b)
	β (95% CI)	
Intercept^a^	1.634 (1.542, 1.724)	5.124 (4.674, 5.607)
Sex^b^	− 0.032 (− 0.162, 0.094)	0.969 (0.850, 1.099)
Previous year relative productivity^c^	0.074 (0.011, 0.139)	1.077 (1.011, 1.149)
Previous year relative productivity × sex^d^	0.011 (− 0.080, 0.096)	1.011 (0.923, 1.101)
	σ^2^ (95% CI)	
Bird ID	0.018 (0.014, 0.022)	1.018 (1.014, 1.022)
Nest ID	0.000 (0.000, 0.000)	1.000 (1.000, 1.000)
Brood ID	0.000 (0.000, 0.000)	1.000 (1.000, 1.000)
Year	0.000 (0.000, 0.000)	1.000 (1.000, 1.000)
Residual^e^	0.178 (0.164, 0.194)	1.195 (1.178, 1.214)
Sample size	n	
Bird ID	165	
Nest ID	158	
Brood ID	215	
Year	15	

Predictor variables are sex (females and males), the relative productivity mean-centred within the previous year (i.e., relative mean-centred number of chicks within the previous year), and their interaction. Parameters were estimated using GLMMs following a Poisson error distribution. Estimated effect sizes and 95% CIs are shown on the latent (a) and data (b) scale. Estimated effect size and 95% CIs were back-transformed from the latent scale using the function “exp” (package base, R Core Team 2024)

^a^Reference category: estimate is the age for females that were spotted for the first time on nests based on the relative productivity mean-centred within the previous year

^b^Estimate is the difference between females (reference) and males (main effect)

^c^Effect the previous year mean-centred productivity of a nest within the previous year (number of chicks)

^d^Estimate is the difference between females (reference) and males in the effects described in the footnote c

^e^Defined as ln(1/exp(βo) + 1) (following Nakagawa & Schielzeth [79])

### Proportion of occupancy

An increment in the proportion of occupancy of a nest during 18 consecutive years was moderately positively (though not significant as 95% CIs for the main effect of Proportion of occupancy overlapped zero; Table 3a) correlated with the age when a bird was spotted for the first time on a nest. The model further suggested that there were no sex differences (as 95% CIs for the interaction Proportion of occupancy × Sex overlapped zero; Table 3a). We thus reran the model to understand the effect of sex and the relative productivity of the nest in the previous year after removing the non-significant proportion of occupancy × Sex interaction term. This model showed that an increase in the mean-centered proportion of occupancy of a nest during 18 consecutive years was positively related to the age of the bird (main effect Proportion of occupancy; Table S3a, Fig. 2c). The back-transformed estimates of our model (Table S3b) showed that the age of the birds with a mean-centered proportion of occupancy during 18 consecutive years was 4.87 years (intercept value Table S3b). Specifically, increasing the mean-centered proportion of occupancy during 18 consecutive years by one, increased the age of the focal bird by a factor of 1.261 (main effect of Proportion of occupancy; Table S3b). Difference in the significance of the mean-centered proportion of occupancy during 18 consecutive years between the models with and without interaction with sex might be due to the number of interacting fixed effects and thus the degrees of freedom.

**Table 3:** Estimated effect sizes and 95% credible intervals (CIs) for predictors of the age when a focal bird was spotted for the first time on a nest based on the proportion of occupancy of the nest during 18 consecutive years

	Age of the focal bird (n = 276 birds)	
	(a)	(b)
	β (95% CI)	
Intercept^a^	1.585 (1.498, 1.665)	4.879 (4.473, 5.286)
Sex^b^	− 0.022 (− 0.137, 0.098)	0.978 (0.872, 1.103)
Proportion of occupancy^c^	0.148 (− 0.128, 0.426)	1.160 (0.880, 1.531)
Sex × proportion of occupancy^d^	0.165 (− 0.234, 0.575)	1.179 (0.791, 1.777)
	σ^2^ (95% CI)	
Bird ID	0.022 (0.018, 0.027)	1.022 (1.018, 1.027)
Nest ID	0.000 (0.000, 0.000)	1.000 (1.000, 1.000)
Brood ID	0.000 (0.000, 0.000)	1.000 (1.000, 1.000)
Year	0.000 (0.000, 0.000)	1.000 (1.000, 1.000)
Residual^e^	0.186 (0.173, 0.202)	1.204 (1.189, 1.224)
Sample size	n	
Bird ID	195	
Nest ID	189	
Brood ID	262	
Year	17	

Predictor variables are sex (females and males), the mean-centred proportion of occupancy of a nest during 18 consecutive years, and their interaction. Parameters were estimated using GLMMs following a Poisson error distribution. Estimated effect sizes and 95% CIs are shown on the latent (a) and data (b) scale. Estimated effect size and 95% CIs were back-transformed from the latent scale using the function “exp” (package base, R Core Team 2024)

^a^Reference category: estimate is the age for females that were spotted for the first time on nests based on the mean-centred proportion of occupancy of a nest during 18 consecutive years

^b^Estimate is the difference between females (reference) and males (main effect)

^c^Effect the mean-centred proportion of occupancy of a nest during 18 consecutive years. Mean value of the occupancy was 0.699

^d^Estimate is the difference between females (reference) and males in the effects described in the footnote c

^e^Defined as ln(1/exp(βo) + 1) (following Nakagawa & Schielzeth [79])

### Type of habitat coverage

**Fig. 3:**
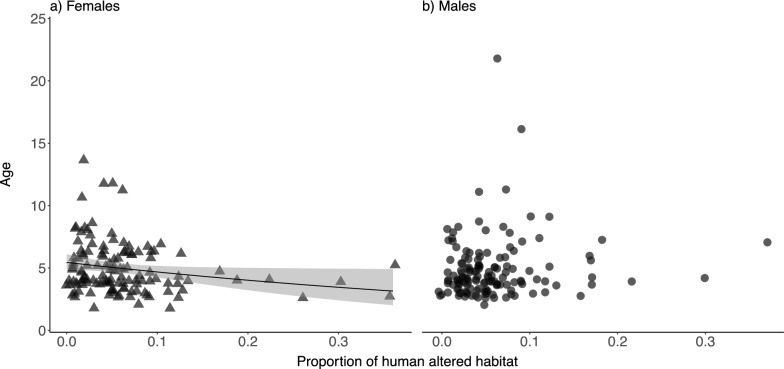
Jitter plots showing the relationships between age of a bird that bred on the nest for the first time (y-axis) and the proportion of habitat with areas altered by humans (x-axis), for females (closed triangles in panel **a**) and males (closed circles in panel **b**), with 95% confidence intervals (grey areas). Each jitter figure represents the age of each individual bird

In females, the proportion of habitat covered by areas altered by humans was negatively related to the age of the bird (main effect Human altered where female was set as reference, Table 4a). We further found sex differences in the age of the bird and the proportion of area altered by humans (interaction Human altered × sex, Table 4). We thus reran the models for females and males separately to understand the effect of the habitat on each sex. The model that included only females suggested that there was a negative effect of the proportion of habitat with areas altered by humans (Table S4b), where the age of the female birds surrounded by areas that were not altered by humans was of 5.20 years (intercept value, table S4b) and a decrease on the age by a factor of 0.211 (main effect Human altered, Table S4b). Nonetheless, we did not detect any effect on males (as 95% CIs for Human altered overlapped zero; Table 4c). Sex differences detected in the model that included both sexes (Table 4a) might have existed because the interactive effects described for females (Table 4b, Fig. 3a) did not exist in males (Table 4c, Fig. 3b). Therefore, this demonstrated that the effects were sex-specific.

**Table 4:** Estimated effect sizes and 95% credible intervals (CIs) for predictors of the age when a focal bird was spotted for the first time on a nest based on the habitat surrounding the nest

	a) Age of the focal bird (n = 276 birds)	b) Age of the focal female (n = 130 females)	c) Age of the focal male (n = 146 males)
	β (95% CI)	β (95% CI)	β (95% CI)
Intercept	1.654 (1.162, 2.144)	1.648 (1.165, 2.102)	1.453 (0.918, 2.010)
Sex	− 0.184 (− 0.930, 0.514)	–	–
*Habitat surface*^*c*^			
Altered by humans	− 1.595 (− 3.067, − 0.008)	− 1.554 (− 3.103, − 0.012)	1.030 (− 0.517, 2.553)
Arable lands	0.034 (− 0.607, 0.660)	0.059 (− 0.509, 0.687)	− 0.044 (− 0.707, 0.588)
Pastures	0.018 (− 0.649, 0.723)	0.016 (− 0.690, 0.728)	0.483 (− 0.636, 1.518)
*Habitat surface* × *Sex*^*d*^			
Altered by humans × Sex	2.622 (0.589, 4.860)	–	–
Arable lands × Sex	− 0.084 (− 0.955, 0.801)	–	–
Pastures × Sex	0.475 (− 0.755, 1.681)	–	–
	σ^2^ (95% CI)	σ^2^ (95% CI)	σ^2^ (95% CI)
Bird ID	0.023 (0.018, 0.027)	0.012 (0.009, 0.015)	0.046 (0.034, 0.059)
Nest ID	0.000 (0.000, 0.000)	0.000 (0.000, 0.000)	0.000 (0.000, 0.000)
Brood ID	0.000 (0.000, 0.000)	–	–
Year	0.000 (0.000, 0.000)	0.000 (0.000, 0.000)	0.000 (0.000, 0.000)
Residual ^e^	0.175 (0.111, 0.272)	0.176 (0.115, 0.272)	0.210 (0.126, 0.336)
Sample size	n		
Bird ID	195	90	105
Nest ID	189	109	117
Brood ID	262	–	–
Year	17	14	16

Predictor variables are sex (females and males), the proportion of habitat surface altered by humans, arable lands, and pastures, and their interactions with sex (only model a). Parameters were estimated using GLMMs following a Poisson error distribution. Estimated effect sizes and 95% CIs are shown on the latent scale

^a^Reference category: estimate is the age for females (models a and b) or males (model c) that were spotted for the first time on nests based on the habitat surface

^b^Estimate is the difference between females (reference) and males (main effect)

^c^Effect the proportion of habitat surface

^d^Estimate is the difference between females (reference) and males in the effects described in the footnote c

^e^Defined as ln(1/exp(βo) + 1) (following Nakagawa & Schielzeth [79])

## Discussion

Our study sheds light on age-based segregation in the occupation of nesting sites, revealing significant correlations between individual age and both nest surface area and previous year nest productivity. While our results on the relationship between age and the proportion of nest occupancy were somewhat inconclusive, they suggest that older birds may prefer nests that are occupied at higher rates. Contrary to our initial expectations, we found no significant relationships between individual age and the prevalence of preferred type of foraging grounds – meadows and pastures or arable lands.

The observed correlation between nest surface area and the bird age aligns with existing research suggesting that larger nests may offer significant advantages in some species [29, 58]. In white stork, Vergara et al. [32] noted that limited nest space can hinder fledging, as chicks require room to exercise their wings—but they also found no consistent relationship between nest size and the number of fledglings, a result echoed in other studies [44].

Nest size may additionally function as a sexually selected trait—an extended phenotype signaling individual quality [59–64]. In species that reuse nests like the white stork, selection may favor the defense rather than construction of nests [32]. Building from scratch is energetically costly and typically occurs only when no suitable nests are available [48]. The association between nest size and age in our study supports the notion that older, more experienced individuals are more likely to secure larger, higher-quality nests. However, prior research in other white stork populations found that while larger nests are occupied earlier, they are not necessarily chosen by older birds [32], though they tend to be selected by more effective breeders that produce larger clutches [32, 44–46]. These patterns may differ in short-distance migratory or sedentary populations in Western Europe and North Africa [65, 66].

In our study, nest surface—but not height—was positively associated with both breeder age and the probability of fledging at least one chick (Text S1, Table S5). This supports two hypotheses proposed by Vergara et al. [32]: that limited surface area may constrain successful rearing of chicks, and that large nests may be monopolized by high-quality individuals. However, we found no relationship between nest surface and the number of fledglings in successful nests. This may result from the specific nature of our dataset, which focuses solely on individuals switching nests—potentially masking the effect of nest quality on lifetime reproductive output. Future studies could examine whether nest size serves primarily as a cue in sexual selection rather than a physical constraint.

Finally, the structural support of the nest (e.g., trees, posts, chimneys, rooftops) may influence nest size potential. Some structures simply cannot support large nests, although variation observed even within the same platform types suggests that experienced individuals may partially overcome these physical limitations.

The correlation between prior nest productivity and bird age suggests that experienced individuals tend to take over nests with a history of higher reproductive success. While previous studies have shown that white storks preferentially return to successful nests [3, 4], our results imply that even when site fidelity is excluded—as in our dataset, which only includes the first recorded use of a nest by a given individual—older birds are still more likely to settle in previously productive locations. This pattern supports the idea that storks may use public information [34, 35] or other quality-related cues when selecting a nest site. Importantly, this result highlights that experienced birds are not only capable of acquiring high-quality nests, but also of using relevant information to do so—even in unfamiliar territories. Interestingly, our findings also suggest that land cover characteristics are either not used as primary cues for settlement, or that both younger and older individuals respond to them similarly in terms of preferences and success in occupying such sites.

While the correlation between the proportion of occupancy and the age of birds was not statistically significant in our study, removing the interaction between sex and proportion of occupancy revealed a significant relationship where older birds preferred nests that were occupied more often. This may suggest that there is high competition over the optimal nests, with preferred nests being occupied at higher rates. The observed pattern hints at possible site fidelity effect where older birds continue to use nests with known histories, potentially independent of nest size or habitat quality [4]. However, this result should be interpreted with caution, as the findings remain inconclusive and rather weak. Additionally, storks are known to prefer reusing nests over building new ones [48] and the proportion of occupancy used in this study—calculated relative to the study period rather than nest existence—may not perfectly reflect this preference. The results show that the age of a female is negatively correlated with human-altered habitat cover, which suggests a preference towards less built-up areas among older females. This pattern implies that younger or less experienced birds are more likely to be relegated to suboptimal nesting sites due to increased competition or habitat scarcity. An alternative explanation could be that less experienced females select nest sites closer to human settlements to take advantage of supplementary feeding opportunities provided by people, whether through intentional feeding or access to waste disposal sites like open landfills. Storks are among opportunistic species known for using different anthropogenic sources of food [67, 68]. This tendency is more pronounced in females, possibly due to their greater investment in reproduction and the need for secure nesting sites to ensure offspring survival.

Notably, older individuals did not choose areas with a higher proportion of pastures more often than younger individuals, and younger individuals were not found in habitats dominated by arable lands more often than older birds. Previous studies suggested that white storks prefer pastures and meadows, while arable lands are rather suboptimal habitats [40, 41, 69, 70]. In this context, our results diverge significantly from ideal despotic distribution theory [9], which posits that subordinate or less experienced individuals, such as younger storks, are pushed into lower-quality habitats by more dominant older individuals. However, recent studies suggest that white storks have adapted to utilize arable lands effectively. Research by Bialas et al. and Kamiński et al. [42, 71] indicated a preference for these habitats. This raises questions about the extent to which the importance of these areas is being underestimated, while adaptation to land-use changes is certainly occurring. Another possible explanation of our findings is that some areas may be critical for territory establishment regardless of individuals age and experience.

Although we found that the effect of some habitat types (e.g., pastures and meadows) on nest-site selection was limited, this may partially stem from lower variability in their availability across the landscape. However, other land-cover types, such as arable land, exhibited substantial variation, and human-altered habitats showed a detectable effect despite lower variance. We acknowledge that uneven variability among habitat categories may influence the strength and detectability of these relationships.

One important factor that we purposely did not incorporate into our models is site fidelity. Previous studies have shown that white storks, like many bird species, exhibit strong site fidelity, with individuals more likely to return to nesting sites where they have bred successfully [3, 4, 72, 73]. Site fidelity is closely linked to age, with older birds more likely to return to previous nesting sites, particularly those associated with high reproductive success. This phenomenon likely interacts with habitat selection, meaning that older, site-faithful storks are able to secure the best nesting sites year after year, further compounding the advantages they gain through experience. This pattern has been observed in other species, such as painted buntings *Passerina ciris*, where high-quality sites are first occupied by new breeders but eventually taken over by site-faithful males [74]. This may also apply to white storks, as this species is known for nest turnover at the beginning of the breeding season [75].

However, due to the strong site fidelity observed in our study population [4], repeated observations of individual storks across multiple nests were rare. As a result, our analyses and interpretations are based on between-individual differences in nest-site selection, not within-individual changes over time. Exploring how individual preferences may evolve with age or breeding experience would require a dedicated longitudinal dataset, which currently remains a limitation. Similarly, as noted earlier, our dataset emphasizes initial nest-site settlement rather than long-term fidelity, and therefore highlights different aspects of habitat selection than studies based on lifetime breeding trajectories. Future studies should focus on the fitness consequences of different settlement decisions across age classes.

Unfortunately, we were unable to determine the age of both members of a breeding pair in most cases. This limitation is noteworthy in the context of scarce studies on age-assortative mating in this species [76], found strong age correlation but no significant effect of age differences between pair members on reproductive success in white storks. This suggests that while storks may pair assortatively by age, age asymmetry per se may not directly influence breeding outcomes. In other species, like the Canada warbler *Cardellina canadensis*, younger males paired with older females tend to reduce reproductive success, possibly because they occupy lower-quality territories [77]. Understanding how the ages of both breeders in a pair interact to influence the choice of territory and reproductive output in white storks could be an important topic for a further study.

Lastly, our study did not account for food subsidies from sources such as landfills or intentional feeding by humans, both of which could significantly influence the overall quality of habitats. These additional food sources may elevate the perceived quality of certain areas, particularly those near human settlements. The white stork is an opportunistic bird species like corvids and potentially explores a variety of anthropogenic food sources [78]. However, this phenomenon seems to be at the developing stage in this population of storks [40, 42]. Nevertheless, future research should incorporate these factors to gain a more accurate understanding of how they affect habitat selection and reproductive success in white storks. Individually marked populations like presented in this study might be a good research setting for testing these effects.

## Conclusion

This study’s implications are consistent with existing literature that underscores the complexity of nesting behavior influenced by both environmental and intrinsic factors. Future research could benefit from integrating longitudinal tracking of individual birds to better understand the causative factors behind nest selection and the differential impacts on reproductive success across genders. Such studies could help clarify the adaptive nature of nest reuse and selection strategies in birds, particularly in rapidly changing environments. In summary, our findings offer important insights into how age and experience shape habitat selection in white storks, while also highlighting the potential impact of age assortative mating, and food subsidies. Addressing these factors in future research will be essential for understanding the full complexity of habitat selection and reproductive success in this species.

## Supplementary Information

Additional file 1.

## Data Availability

The datasets used and/or analysed during the current study are available from the corresponding author on reasonable request.
