# Supplementary material for: Age-related nest-site segregation in a solitary nesting population of white stork Ciconia Ciconia
Source: Front Zool. 2025 Aug 4;22:19. doi: 10.1186/s12983-025-00574-2 (PMC12323261; doi:10.1186/s12983-025-00574-2)
Supplement: Supplementary file 1 — Additional file 1. [file 12983_2025_574_MOESM1_ESM.pdf]

Supplementary Material

Age-related nest-site segregation in a solitary nesting population of white stork *Ciconia Ciconia*

Joanna T. Bialas[1]*, Irene Gaona-Gordillo[1,2], Lukasz Dylewski[1], Marcin Tobolka[1]

[1]Department of Zoology, Poznań University of Life Sciences, Wojska Polskiego 71C, 60-625 Poznań, Poland

[2]Behavioural Ecology, Department of Biology, Ludwig Maximilians University of Munich, Planegg-Martinsried, Germany

*Correspondence: jtwozna@gmail.com

Joanna T. Bialas: https://orcid.org/0000-0002-0683-4001

Irene Gaona-Gordillo: https://orcid.org/0000-0001-5437-5754

Łukasz Dylewski: https://orcid.org/0000-0002-1370-7625

Marcin Tobolka: https://orcid.org/0000-0002-4989-1524

Table of contents

Table S1. 3

Table S2 4

Table S3. 5

Table S4 6

Text S1 7

Table S5 8

References 9

Table S1. Estimated effect sizes and 95% credible intervals (CIs) for predictor of the age when a focal bird was spotted for the first time in a nest based on the height and the surface of the nest. Predictor variables are sex (females and males), the mean-centred height of the nest (in m), and the mean-centred surface of the nest (in m^2). Parameters were estimated using GLMMs following a Poisson error distribution. Estimated effect sizes and 95% CIs are shown on the latent (a) and data (b) scale. Estimated effect size and 95% CIs were back-transformed from the latent scale using the function "exp" (package base, R Core Team 2024).

	Age of the focal bird (n = 152 birds)	
	(a)	(b)
	β (95% CI)	
Intercept [a]	1.632 (1.522, 1.744)	5.114 (4.581, 5.720)
Sex [b]	-0.036 (-0.177, 0.105)	0.965 (0.838, 1.111)
Dimensions of the nest		
Height [c]	0.130 (-0.121, 0.382)	1.139 (0.886, 1.465)
Surface [d]	0.147 (0.014, 0.281)	1.158 (1.014, 1.324)
	σ^2 (95% CI)	
Bird ID	0.000 (0.000, 0.000)	1.000 (1.000, 1.000)
Nest ID	0.000 (0.000, 0.000)	1.000 (1.000, 1.000)
Brood ID	0.000 (0.000, 0.000)	1.000 (1.000, 1.000)
Year	0.010 (0.004, 0.017)	1.010 (1.004, 1.017)
Residual [e]	0.179 (0.161, 0.197)	1.196 (1.175, 1.218)

[a] Reference category: estimate is the age for females that were spotted for the first time on nests of average height and average surface.
[b] Estimate is the difference between females (reference) and males (main effect).
[c] Effect of nests with mean-centred height (in m). Mean height of the nest was 0.663 m.
[d] Effect of nests with mean-centred surface (in m^2). Mean surface of the nest was 1.825 m^2.
[e] Defined as $\ln(1/\exp(\beta_o)+1)$ (following Nakagawa & Schielzeth, 2010).

Table S2. Estimated effect sizes and 95% credible intervals (CIs) for predictor of the age when a focal bird was spotted for the first time on a nest based on the relative productivity of a nest within the previous year. Predictor variables are sex (females and males) and the relative productivity mean-centred within the previous year (i.e., relative mean-centred number of chicks within the previous year). Parameters were estimated using GLMMs following a Poisson error distribution. Estimated effect sizes and 95% CIs are shown on the latent (a) and data (b) scale. Estimated effect size and 95% CIs were back-transformed from the latent scale using the function “exp” (package base, R Core Team 2024).

	Age of the focal bird (n = 222 birds)	
	(a)	(b)
	β (95% CI)	
Intercept [a]	1.632 (1.546, 1.719)	5.114 (4.693, 5.579)
Sex [b]	-0.032 (-0.162, 0.096)	0.969 (0.850, 1.101)
Previous year relative productivity [c]	0.079 (0.034, 0.122)	1.082 (1.035, 1.130)
	σ^2 (95% CI)	
Bird ID	0.018 (0.014, 0.022)	1.018 (1.014, 1.022)
Nest ID	0.000 (0.000, 0.000)	1.000 (1.000, 1.000)
Brood ID	0.000 (0.000, 0.000)	1.000 (1.000, 1.000)
Year	0.000 (0.000, 0.000)	1.000 (1.000, 1.000)
Residual [d]	0.179 (0.165, 0.193)	1.196 (1.179, 1.213)

[a] Reference category: estimate is the age for females that were spotted for the first time on nests based on the previous year’s mean-centred productivity on a nest.

[b] Estimate is the difference between females (reference) and males (main effect).

[c] Effect the previous year mean-centred productivity of a specific nest (number of chicks).

[d] Defined as ln(1/exp(βo)+1) (following Nakagawa & Schielzeth, 2010).

Table S3. Estimated effect sizes and 95% credible intervals (CIs) for predictor of the age when a focal bird was spotted for the first time on a nest based on the proportion of occupancy of the nest during 18 consecutive years. Predictor variables are sex (females and males) and the mean-centred proportion of occupancy of a nest during 18 consecutive years. Parameters were estimated using GLMMs following a Poisson error distribution. Estimated effect sizes and 95% CIs are shown on the latent (a) and data (b) scale. Estimated effect size and 95% CIs were back-transformed from the latent scale using the function "exp" (package base, R Core Team 2024).

	Age of the focal bird (n = 276 birds)	
	(a)	(b)
	β (95% CI)	
Intercept [a]	1.582 (1.501, 1.665)	4.865 (4.486, 5.286)
Sex [b]	-0.018 (-0.133, 0.096)	0.982 (0.875, 1.101)
Proportion of occupancy [c]	0.232 (0.036, 0.434)	1.261 (1.037, 1.543)
	σ^2 (95% CI)	
Bird ID	0.022 (0.018, 0.027)	1.022 (1.018, 1.027)
Nest ID	0.000 (0.000, 0.000)	1.000 (1.000, 1.000)
Brood ID	0.000 (0.000, 0.000)	1.000 (1.000, 1.000)
Year	0.000 (0.000, 0.000)	1.000 (1.000, 1.000)
Residual [d]	0.187 (0.173, 0.201)	1.206 (1.189, 1.223)

[a] Reference category: estimate is the age for females that were spotted for the first time on nests based on the mean-centred proportion of occupancy of a nest during 18 consecutive years.

[b] Estimate is the difference between females (reference) and males (main effect).

[c] Effect the mean-centred proportion of occupancy of a nest during 18 consecutive years. Mean value of the occupancy was 0.699.

[d] Defined as ln(1/exp(βo)+1) (following Nakagawa & Schielzeth, 2010).

Table S4. Estimated effect sizes and 95% credible intervals (CIs) for predictor of the age when a focal bird was spotted for the first time on a nest based on the habitat surrounding the nest. Predictor variables are sex (females and males), the proportion of habitat surface altered by humans, arable land, and pastures, and their interactions (only model a). Parameters were estimated using GLMMs following a Poisson error distribution. Estimated effect size and 95% CIs were back-transformed from the latent scale using the function "exp" (package base, R Core Team 2024). Parameters are thus shown in the data scale.

	a) Age of the focal bird (n = 276 birds)	b) Age of the focal female (n = 130 females)	c) Age of the focal male (n = 146 males)
	β (95% CI)	β (95% CI)	β (95% CI)
Intercept	5.228 (3.196, 8.534)	5.197 (3.206, 8.183)	4.276 (2.504, 7.463)
Sex	0.832 (0.395, 1.672)	-	-
Habitat surface [c]			
Altered by humans	0.203 (0.047, 0.992)	0.211 (0.045, 0.988)	2.801 (0.596, 12.846)
Arable lands	1.035 (0.545, 1.935)	1.061 (0.601, 1.988)	0.957 (0.493, 1.800)
Pastures	1.018 (0.523, 2.061)	1.016 (0.502, 2.071)	1.621 (0.529, 4.563)
Habitat surface × Sex [d]			
Altered by humans × Sex	13.763 (1.802, 129.024)	-	-
Arable lands × Sex	0.919 (0.385, 2.228)	-	-
Pastures × Sex	1.608 (0.470, 5.371)	-	-
	σ^2 (95% CI)	σ^2 (95% CI)	σ^2 (95% CI)
Bird ID	1.023 (1.018, 1.027)	1.012 (1.009, 1.015)	1.047 (1.035, 1.061)
Nest ID	1.000 (1.000, 1.000)	1.000 (1.000, 1.000)	1.000 (1.000, 1.000)
Brood ID	1.000 (1.000, 1.000)	-	-
Year	1.000 (1.000, 1.000)	1.000 (1.000, 1.000)	1.000 (1.000, 1.000)
Residual [e]	1.191 (1.117, 1.313)	1.192 (1.122, 1.313)	1.234 (1.134, 1.399)

[a] Reference category: estimate is the age for females (models a and b) or males (model c) that were spotted for the first time on nests based on the habitat surface.

[b] Estimate is the difference between females (reference) and males (main effect).

[c] Effect the proportion of habitat surface.

[d] Estimate is the difference between females (reference) and males in the effects described in the footnote c.

[e] Defined as ln(1/exp(βo)+1) (following Nakagawa & Schielzeth, 2010).

Text S1. We analysed whether the hight and the surface of the nest had an effect on the success of the brood. To avoid analytical complexity, we first, analyzed the binary probability of producing chicks (n = 140 broods), and then, for the nests with at least one chick, we analyzed the effects on the number of chicks (n = 123 broods). Models were fitted with a binomial error distribution with the logit link function and a Poisson error distribution with a log-link function, respectively. For further explanation see Dingemanse et al. (2020). Both models fitted the mean-centered height and mean-centered surface as covariates. We further fitted random intercepts for the nest identity and year (for further details see main text). Our models showed that nests with an increased surface were more likely to have at least one chick (main effect of Surface; Table 5a). Nonetheless, this effect was not observed on the number of chicks produced on successful nests (main effect of Surface, Table 5b).

Table S5. Estimated effect sizes and 95% credible intervals (CIs) for predictor of (a) the binary probability of producing chicks and (b) the number of chicks. Predictor variables are the mean-centred height of the nest (in m) and the mean-centred area of the nest (in m^2). Parameters were estimated using GLMMs following a binomial (model a) and Poisson (model b) error distributions. Estimated effect sizes and 95% CIs are shown on the latent scale.

	(a) Binary probability of producing chicks (n = 140 broods)	(b) Number of chicks (n = 123 broods)
	β (95% CI)	
Intercept [a]	2.270 (1.573, 2.960)	0.900 (0.787, 1.012)
Dimensions of the nest		
Height [c]	1.535 (-0.524, 3.615)	-0.039 (-0.429, 0.329)
Surface [d]	1.452 (0.036, 2.913)	0.102 (-0.098, 0.298)
	σ^2 (95% CI)	
Nest ID	0.000 (0.000, 0.000)	0.000 (0.000, 0.000)
Year	0.185 (0.071, 0.368)	0.000 (0.000, 0.000)
Residual [e]	$\pi^{2/3}$	0.341 (0.310, 0.375)
Sample size	n	
Nest ID	115	104
Year	14	14

[a] Reference category: estimate is for bird breeding in nests with average height and average surface.

[b] Effect of nests with mean-centred height (in m). Mean height of the nest was 0.663 m.

[c] Effect of nests with mean-centred area (in m2). Mean area of the nest was 1.825 m2.

[d] Residual variance fixed to $\pi^{2/3}$ (following Nakagawa & Schielzeth, 2010) for the models of the binary probability of producing chicks (model a) or defined as ln(1/exp(βo)+1) (following Nakagawa & Schielzeth, 2010) for the model of number of chicks (model b).

References

Dingemanse NJ, Wright J. 2020. Criteria for acceptable studies of animal personality and behavioural syndromes. Ethology. 126(9):865–869. doi:10.1111/eth.13082.

Nakagawa S, Schielzeth H. 2010. Repeatability for Gaussian and non-Gaussian data: a practical guide for biologists. Biol Rev. 85(4):935–956. doi:10.1111/j.1469-185X.2010.00141.x.
